# The Small RNA MicF Represses ObgE and SeqA in *Escherichia coli*

**DOI:** 10.3390/microorganisms12122397

**Published:** 2024-11-22

**Authors:** Aaron Y. Stibelman, Amy Y. Sariles, Melissa K. Takahashi

**Affiliations:** 1 Department of Biology, California State University Northridge, Northridge, CA 91330, USA; as380@rice.edu (A.Y.S.); amy.sariles.4@my.csun.edu (A.Y.S.); 2 Department of Biosciences, Rice University, Houston, TX 77005, USA

**Keywords:** RNA, gene regulation, *Escherichia coli*

## Abstract

Small regulatory RNAs (sRNA) have been shown to play a large role in the management of stress responses in *Escherichia coli* and other bacteria. Upon fluctuations in nutrient availability and exposure to antimicrobials and superoxide-generating agents, the MicF sRNA in *E. coli* has been shown to regulate a small set of genes involved in the management of membrane permeability. Currently, it is unknown whether MicF acts on other processes to mediate the response to these agents. Using an sRNA interaction prediction tool, we identified genes in *E. coli* that are potentially regulated by MicF. Through subsequent analysis using a sfGFP-based reporter–gene fusion, we have validated two novel targets of MicF regulation: ObgE, a GTPase crucial for chromosome partitioning, and SeqA, a negative modulator of DNA replication. Importantly, the interaction between MicF and these target mRNAs is contingent upon the presence of the RNA chaperone protein, Hfq. Furthermore, our findings affirm the role of MicF’s conserved 5’ seed pairing region in initiating these regulatory interactions. Our study suggests that, beyond its established role in membrane permeability management, MicF exerts control over chromosome dynamics in response to distinct environmental cues, implicating a more multifaceted regulatory function in bacterial stress adaptation.

## 1. Introduction

Bacteria encounter a wide range of pressures in their natural environment such as nutrient availability, oxidative stress, and the presence of antimicrobials. Small regulatory RNAs (sRNAs) aid in the adaptation to these conditions by enabling bacteria to swiftly transition between different physiological states [1,2]. These regulatory RNAs also allow bacteria to efficiently allocate resources and maximize their survival in diverse environmental niches. sRNAs modulate the expression of *trans*-encoded mRNA by base pairing at locations near the 5′ untranslated region (UTR). The hybridization of sRNAs may alter mRNA translation directly by affecting ribosome accessibility or indirectly by modifying mRNA stability [3,4,5]. An important aspect of sRNA-based gene regulation is that it occurs through imperfect base-pairing interactions that allow a single sRNA to act with regulatory plasticity, targeting genes across diverse pathways, linking different biological processes, and facilitating connections within unique cellular networks [2,5,6].

MicF was amongst the earliest chromosomally encoded sRNAs discovered and initially was only associated with the repression of the nonspecific outer membrane protein, OmpF [7,8]. MicF is a 93-nucleotide sRNA with a well-studied secondary structure [9]. MicF’s conservation across γ-proteobacteria [10,11] underscores its pivotal role in responding to various extracellular stresses. MicF expression is activated by the transcription factors Rob, MarA, and SoxS, and repressed by H-NS and the leucine-responsive transcription factor, Lrp [12]. In response to antibiotics or oxidative stress, among other things, MicF reduces outer membrane permeability, enabling survival [13,14,15,16]. Conversely, under nutrient-limiting conditions, MicF expression is suppressed to maximize OmpF production [17]. More recently, additional targets of MicF regulation in *Escherichia coli* were identified, including the mRNAs *lrp*, *cpxR*, *phoE*, and *oppA* [18,19]. These genes expanded MicF’s role in membrane permeability management and suggested a larger role for MicF in metabolism via repression of Lrp, which is responsible for the regulation of approximately 10% of genes in *E. coli* [20].

To further explore the regulatory plasticity of MicF, we used sRNA target prediction tools to identify new candidates for MicF regulation. Using mRNA-sfGFP fusions, Western blot analysis, and cell growth experiments, we identify two additional targets of MicF regulation, *obgE* and *seqA*. The repression of both genes by MicF suggests two additional roles for MicF regarding chromosome replication during conditions of nutrient abundance and oxidative stress.

## 2. Materials and Methods

### 2.1. Growth Conditions

All strains were grown in Lysogeny broth (LB), LB agar (1.5%) plates, MOPS EZ Rich Defined Media (Teknova M2105, Hollister, CA, USA), or M9 at 37 °C. The M9 media contained: 1 × M9 salts, 1 mM thiamine hydrochloride, 0.4% glycerol, 0.2% casamino acids, 2 mM magnesium sulfate, and 0.1 mM calcium chloride. When necessary, media was supplemented with antibiotics (carbenicillin: 100 μg/mL, chloramphenicol: 34 μg/mL, kanamycin: 50 μg/mL).

### 2.2. Strains

The *E. coli* strains used in this study are listed in Table S1. Deletion strains were constructed using the lambda red recombination method [21]. DNA fragments containing a Kan^R^ cassette flanked by two FRT sites were amplified from pKD4 with the appropriate flanking sequences and electroporated into BW25113 for the construction of Δ*micF* and rne-131. The construction of the C-terminal 3xFLAG-tagged strains were carried out similarly using the following modification. The DNA fragments contained a 3xFLAG tag followed by a FRT flanked Cm^R^ cassette. DNA fragments were PCR amplified and electroporated into the Δ*micF* strain. Removal of Kan^R^ or Cm^R^ was achieved using the helper plasmid pCP20 following Datsenko and Wanner [21]. All deletions and insertions were confirmed using PCR and Sanger DNA sequencing. The primers used for creating the deletion strains or 3xFLAG tag strains are listed in Table S1.

### 2.3. Plasmids

All plasmids used in this study are listed in Table S3, with key sequences found in Table S2. Plasmids were constructed using inverse PCR or Gibson assembly. Gene sequences were PCR amplified from purified genomic DNA. New England Biolabs (Ipswich, MA, USA) Turbo *E. coli* cells was used for the transformation of constructed plasmids. All plasmids were verified using Sanger DNA sequencing.

### 2.4. CopraRNA and TargetRNA2

The web-accessible programs CopraRNA and TargetRNA2 were used to identify target genes regulated by MicF in *E. coli*. The organisms included in the CopraRNA query were *E. coli* NC 000913, *Citrobacter koseri* NC009792, *Citrobacter rodentium* NC_013716, *Escherichia fergusonii*, NC 011740, and *Salmonella* enterica subsp. enterica serovar *Typhimurium* NC 003197. The CopraRNA program was run under default conditions. The CopraRNA default consists of sequences extracted around the start codon (200 nucleotides upstream, 100 nucleotides downstream), a dynamic setting for *p*-value, and no *p*-value filtering or consensus prediction. For the TargetRNA2, the MicF sequence was obtained from Genbank NC_000913 and screened against the genome of *E. coli* str. K-12 substr. MG1655.

### 2.5. Fluorescence Measurement and Culturing Conditions

Plasmids were transformed into chemically competent *E. coli* strains, plated on LB-agar plates containing chloramphenicol and carbenicillin, and incubated overnight at 37 °C. Individual colonies from each condition were inoculated into 300 μL of LB with corresponding antibiotics in a 2 mL 96-well block (Corning Costar 3961, Corning, NY, USA) sealed with a breathe-easier membrane (USA Scientific 9126-2100, Madison, FL, USA) and grown for 17 h overnight at 37 °C while shaking at 100 rpm (Labnet Vortemp 56, Edison, NJ, USA). Four microliters of the overnight culture were added to 296 μL of MOPS media that was pre-warmed at 37 °C for 30 min. The cultures were grown under the same conditions as above for 2.5 or 3 h depending on the strain used. This resulted in cultures with an OD_600_ of 1–2.2. One hundred microliters of each culture were transferred to a 96-well plate (Corning Costar 3631, Corning, NY, USA), and OD_600_ and bulk sfGFP fluorescence (485 nm excitation, 520 nm emission) were measured using a Biotek Synergy H1 plate reader. Each experiment included two sets of controls: a media blank and *E. coli* transformed with control plasmids. The OD_600_ and FL for each culture were first corrected by subtracting the mean value of the media blank. The ratio of the corrected FL and OD_600_ (FL/OD_600_) was calculated for each culture. The *E. coli* cultures transformed with control plasmids were used to correct for autofluorescence. The average FL/OD_600_ from the control cultures was subtracted from FL/OD_600_ from each condition.

For experiments assessing endogenously expressed MicF, reporter plasmids were transformed into chemically competent *E. coli* strains, plated on LB-agar plates containing chloramphenicol, and incubated overnight at 37 °C. Individual colonies from each condition were inoculated into 300 μL of LB or M9 and grown overnight as above. Subcultures grown in LB were grown for 2.5 h, while subcultures grown in M9 were grown for 4 h.

### 2.6. Determining Cell Doubling Time

*E. coli* strains were isolated on LB-agar plates containing appropriate antibiotics if necessary. Six individual colonies from each condition were inoculated into 300 μL of LB with corresponding antibiotics, if necessary, in a 2 mL 96-well block (Costar 3961) sealed with a breath-easier membrane and grown for 17 h overnight at 37 °C while shaking at 100 rpm (Labnet Vortemp 56). Four microliters of the overnight culture were added to 296 μL of LB (no antibiotics for all strains) and grown under the same conditions for one hour. From that point, 2 μL from each culture was removed every 20 min and used to determine the CFU/mL via dilutions and plating. The doubling time for an individual colony was determined by taking the log base 2 of each CFU/mL value, then determining the slope log_2_(CFU/mL) vs. time and taking the inverse of that slope value. Doubling times were averaged across the six colonies. For the Δ*micF* pMicF pSeqA condition, *seqA* was transcribed from the inducible promoter p_Lux_ [22] and induced with 10 μM *N*-acyl homoserine lactone (Cayman Chemical 10011207, Ann Arbor, MI, USA).

### 2.7. Protein Purification and Western Blot

*E. coli* cultures were grown overnight at 37 °C while shaking at 275 rpm in LB supplemented with the appropriate antibiotics. Cells were diluted 100-fold in LB and grown to an OD_600_ of approximately 0.5. Cell pellets corresponding to 1 mL of each culture were resuspended using 4× Laemmli buffer and heated for 4 min at 95 °C. For each sample, 10 μL was separated on a 10% SDS-PAGE gel and transferred to a 0.2 μM PVDF membrane (Sigma Aldrich 0301004001, St. Louis, MO, USA) at 40 mA overnight at 4 °C with stirring. Protein expression was visualized using monoclonal anti-FLAG antibodies and HRP (ThermoFisher Scientific MA1-91878-HRP, Waltham, MA, USA) and developed using a Pierce Fast Western Kit SuperSignal West Pico Mouse (ThermoFisher Scientific 35060, Waltham, MA, USA). Blots were developed in SuperSignal West Pico Working Solution for five minutes and imaged using an iBright 1500 (ThermoFisher Scientific, Waltham, MA, USA). The images were processed and quantified using Image J (https://imagej.net/ij/ accessed on 29 October 2024, version 1.54g) software. The intensities of the MicF target protein bands were normalized with the intensities of the corresponding total protein visualized via Ponceau S staining.

### 2.8. Cell-Free Protein Expression to Determine Hfq Dependency

PURExpress (New England Biolabs, E6800, Ipswich, MA, USA) reactions were prepared according to manufacturer’s protocol. Plasmids were provided at the following concentrations: mRNA::sfgfp (1 nM), MicF (4 nM), and Hfq (4 nM). SUPERase-In RNase inhibitor (Thermo Fisher Scientific AM2696, Waltham, MA, USA) was added to each reaction (0.25 μL/10 μL reaction). Eight microliters of each reaction was added to a 384-well plate (Thermo Scientific 142761, Waltham, MA, USA), covered with a seal (VWR 60941-078, Radnor, PA, USA), and placed on a Biotek SynergyH1 plate reader. The temperature was controlled at 37 °C, and sfGFP fluorescence was measured from the bottom of the plate every five minutes (485 nm excitation, 520 nm emission).

## 3. Results

### 3.1. Identification of MicF Regulated mRNA Targets

To identify novel *E. coli* mRNA as candidates for regulation by MicF, we utilized two sRNA target prediction tools, CopraRNA [23] and TargetRNA2 [24]. Both tools have previously been shown to accurately predict in vivo mRNA targets of bacterial sRNAs by generating RNA-RNA interactions after accounting for the intramolecular accessibility of each RNA and the phylogenetic conservation of each interaction. When evaluating the top results from both algorithms (Tables S4 and S5), only CopraRNA predicted known targets of MicF regulation in *E. coli* (*ompF*, *lrp*, *oppA*). Therefore, we restricted our selection of candidate mRNA targets to those predicted by the CopraRNA algorithm. We selected five of the top 10 hits (*obgE*, *seqA*, *hofQ*, *mgrB*, *hypB*) for experimental validation by excluding those that have previously been validated (*lrp* [18], *oppA* [19], *ompF* [25]), invalidated (*murG* [18]), or had uncharacterized function (*ysaB*). We cross-referenced the five candidate mRNAs with published experimental data sets and found that none of the targets were identified using RIL-seq (RNA interaction by ligation and sequencing) [26], while two of the targets (*obgE* and *seqA*) were identified using MAPS (MS2 affinity purification coupled with RNA sequencing) [19].

To evaluate MicF’s ability to regulate the mRNA targets predicted by CopraRNA, we built translational fusions of the mRNAs with superfolder green fluorescent protein (sfGFP) following the work of Urban and Vogel [27] and Corcoran et al. [11]. When testing an sRNA’s ability to regulate an mRNA, it is common to truncate the coding sequence (CDS) of the target when fusing it to the reporter protein [27,28]. As the length of the fused coding sequence could impact both sRNA binding and sfGFP protein stability, we built four variants for each target that included either 5, 10, 20, or 40 codons downstream of the predicted MicF interaction site. In the case of *mgrB*, a maximum of only 34 codons were included due to the small size of its CDS. For the predicted monocistronic targets (*seqA* and *mgrB*), the entire 5’ UTR was incorporated into the fusion. For targets predicted within an operon (*obgE*, *hofQ*, and *murG*), a truncated *lacZ* sequence was placed upstream of the target mRNA to mimic the transcription and translation of a polycistronic mRNA [27]. MicF was predicted to interact with *obgE* downstream of the stop codon of the previous gene in its operon, thus the stop codon was placed at the end of the *lacZ* sequence, followed by the *obgE* 5’ UTR. For *hofQ* and *murG*, MicF was predicted to interact with the sequence within the CDS of the previous gene, thus the sequence starting 30 nucleotides upstream of the predicted interaction site was fused to the *lacZ* sequence.

**Figure 1:**
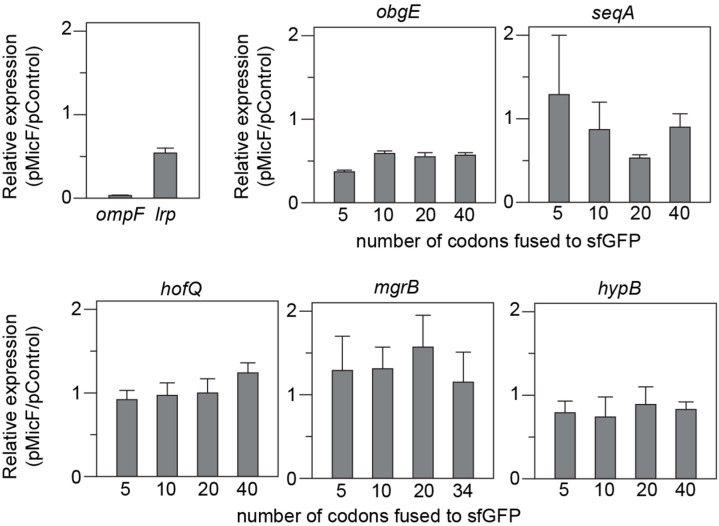
Regulation of mRNA::sfGFP reporter gene fusions by MicF. Target mRNA sequences were cloned as sfGFP fusions on a low-copy vector (pSC101) and transcribed from the constitutive promoter J23118. Four fusions were built for each target, with either 5, 10, 20, or 40 codons beyond the predicted MicF binding site fused to sfGFP. mRNA::sfGFP fusions were transformed into *E. coli* BW25113 with either a MicF overexpression plasmid (pMicF) or a control (pControl) on a medium-copy vector (p15A) also transcribed from the J23118 promoter. sfGFP fluorescence and OD_600_ was measured for each condition. Each bar represents the ratio of average fluorescence/optical density (FL/OD_600_) between cells harboring pMicF and pControl. mRNA::sfGFP fusions for *ompF* and *lrp* were included as references. Error bars were calculated from six biological replicates (see Figure S1).

The expression of each candidate mRNA target was evaluated in the presence and absence of MicF by transforming each mRNA::sfGFP plasmid into *E. coli* BW25113 along with either a control plasmid (pControl) or one that constitutively transcribes MicF (pMicF). The known targets *ompF::sfGFP* and *lrp::sfGFP* were included for comparison. Of the five genes tested, *obgE::sfGFP* and *seqA::sfGFP* had variants that were repressed at least two-fold by MicF (Figure 1). The 20-codon fusion for *seqA* was the only one of its variants that was repressed by MicF. Upon folding the *seqA* fusion mRNA’s in NUPACK [29,30], we discovered that the other three fusion lengths were predicted to form secondary structures that would block the region MicF was predicted to bind (Figure S2). Although several *mgrB::sfGFP* variants showed increased expression in the presence of MicF, we chose to only pursue targets that exhibited at least a two-fold change in expression. The *obgE* 5-codon and the *seqA* 20-codon fusions were used for all subsequent experiments.

**Figure 2:**
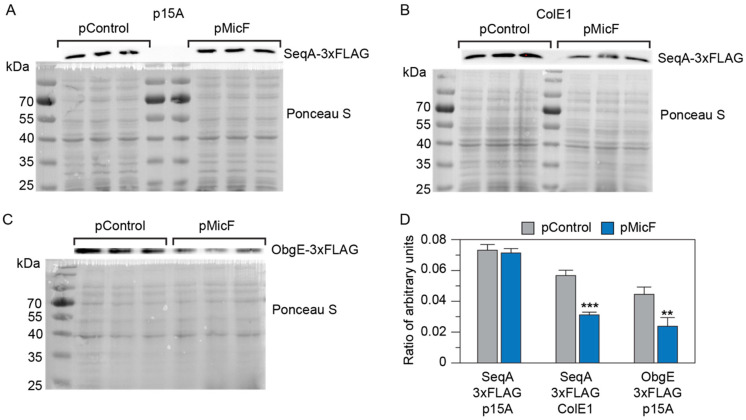
MicF represses ObgE and SeqA expression. Western blot analysis of SeqA-3xFLAG (**A**,**B**) or ObgE-3xFLAG (**C**) protein expressed in *E. coli* BW25113 Δ*micF* transformed with either a MicF overexpression plasmid (pMicF) or control (pControl) on a medium-copy vector (p15A) or high-copy vector (ColE1), as indicated. (**D**) Protein bands from (**A**–**C**) were analyzed via Image J and normalized to total protein in each lane determined from the Ponceau S stain. Bars show mean values and error bars represent the standard deviations of the three replicates. A two-tailed *t*-test was used to compare pControl and pMicF conditions. The significance is marked by asterisks above the pMicF bars indicating *p* < 0.01 (**) or *p* < 0.001 (***).

To confirm that MicF represses the expression of ObgE and SeqA in vivo, we performed a Western blot analysis by chromosomally inserting a 3xFLAG epitope at the C-terminal end of either the *obgE* or *seqA* CDS in their native locus and used anti-FLAG antibodies to observe the protein levels in a strain of *E. coli* with the MicF gene deleted. The production of ObgE::3xFLAG and SeqA::3xFLAG was compared between a strain complemented with MicF from a medium-copy plasmid (pMicF) and a strain transformed with a control plasmid (pControl). The Western blot analyses confirmed that MicF negatively regulates ObgE, as its expression was decreased in the presence of MicF (Figure 2C,D). For SeqA, no significant difference was observed when MicF was overexpressed from the medium-copy plasmid (Figure 2A,D). However, a decrease in expression was observed when MicF was overexpressed from a high-copy plasmid (Figure 2B,D).

### 3.2. The RNA Chaperone Protein Hfq Is Required for MicF’s Inhibition of obgE and seqA

**Figure 3:**
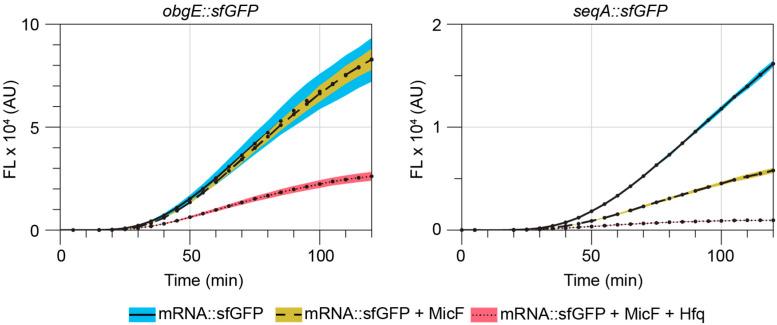
MicF’s regulation of *obgE* and *seqA* is dependent on the chaperone protein Hfq. Cell-free protein expression reactions were run with *obgE::sfgfp* or *seqA::sfgfp* plasmids with or without plasmids expressing MicF and Hfq. Shaded regions represent the standard deviations from three independent reactions calculated at each time point.

Due to their intrinsically weak posttranscriptional interactions with mRNA, many sRNA require the RNA chaperone protein Hfq to more stably interact with their targets [2,4]. Hfq supports RNA-RNA interactions by binding to both molecules and causing an increase in their local concentrations [31,32]. Additionally, as Hfq binds to each RNA, it facilitates the melting of intramolecular secondary structures, further promoting and identifying the correct sRNA-mRNA hybridization [33,34,35]. Since Hfq is required for regulation of MicF’s known targets [11,27], we investigated its necessity for the repression of *obgE* and *seqA*. We transformed the mRNA::sfGFP fusions with pMicF or pControl into a Δ*hfq* strain of *E. coli* BW25113. In the Hfq deleted strain, MicF’s repression of both *obgE::sfGFP* and *seqA::sfGFP* was completely lost (Figure S3). However, it has been shown that MicF is unstable in a Δ*hfq* strain [27,36]. Therefore, we utilized a cell-free protein expression system (PURExpress) that is manufactured from purified components [37] to assess the importance of Hfq. RNA degradasome components are not included in the system and the degradation of RNA is further limited with the addition of an RNase inhibitor (see Section 2). Plasmids for each mRNA::sfGFP construct were expressed in reactions with or without plasmids expressing MicF and Hfq. Repression of *obgE::sfGFP* was only observed when both MicF and Hfq were present in the reaction (Figure 3). Repression of *seqA::sfGFP* was observed with MicF alone and was further enhanced by the presence of Hfq (Figure 3). Together these results suggest that, like its other targets, MicF’s inhibition of *obgE* and *seqA* expression is dependent on the presence of Hfq.

### 3.3. MicF’s 13-Nucleotide Seed Pairing Region Is Required for the Repression of obgE and seqA

**Figure 4:**
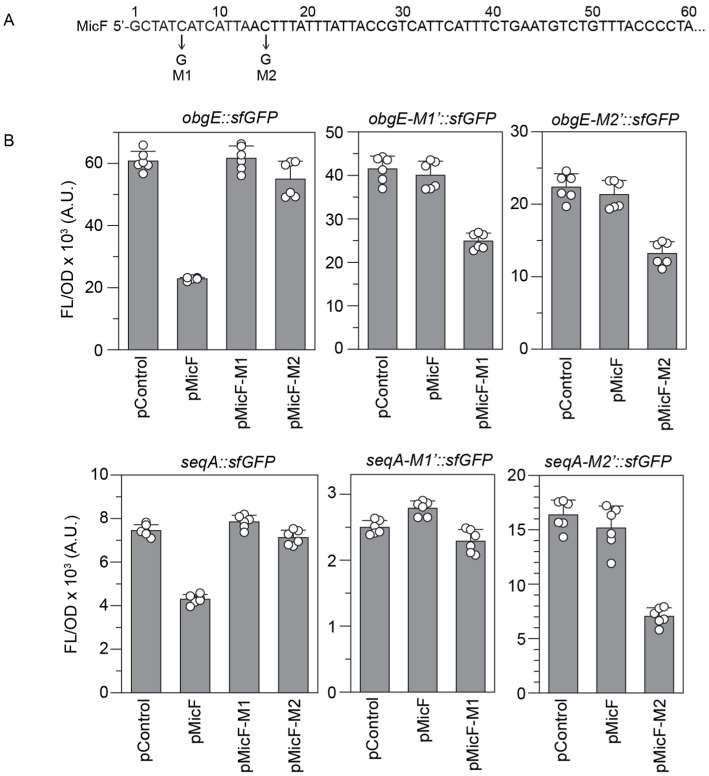
Compensatory mutations confirm MicF binding to *obgE* and *seqA*. (**A**) The sequence for the 5′ end of MicF, indicating the C to G mutations made for MicF-M1 (at C6) and MicF-M2 (at C15). (**B**) Bars show mean values of fluorescence/optical density (FL/OD_600_) from cells with *obgE::sfGFP*, *seqA::sfGFP*, or their mutants in the presence of a control plasmid (pControl) or one that overexpresses a MicF variant. Plasmids were transformed into *E. coli* BW25113. Error bars represent the standard deviations of six biological replicates, shown as open circles.

To confirm that the regulation of *obgE* and *seqA* was occurring due to the binding of MicF, we introduced two different mutations into the MicF sequence along with compensatory mutations to the predicted binding regions within the *obgE::sfGFP* and *seqA::sfGFP* constructs. MicF-M1 changed the sixth nucleotide of MicF from a C to a G, while MicF-M2 changed the fifteenth nucleotide of MicF from a C to a G (Figure 4A). Repression of *obgE::sfGFP* and *seqA::sfGFP* was no longer observed in the presence of pMicF-M1 or pMicF-M2. For *obgE*, both mutant targets (*obgE-M1’::sfGFP* and *obgE-M2’::sfGFP*) were not repressed by wild-type MicF but were repressed by the respective MicF mutant. For *seqA*, the M2 mutant was not repressed by wild-type MicF and was repressed by MicF-M2. However, *seqA-M1’::sfGFP* was not repressed by either wild-type MicF or MicF-M1 (Figure 4B). We note that the fluorescence from cells expressing the *seqA-M1’::sfGFP* construct along with pControl was approximately 2.5 times lower than the *seqA::sfGFP* construct, and the predicted secondary structure of the *seqA-M1’::sfgfp* mRNA indicated that the C to G mutation may induce a structure that sequesters the availability of the predicted MicF binding region (Figure S4).

**Figure 5:**
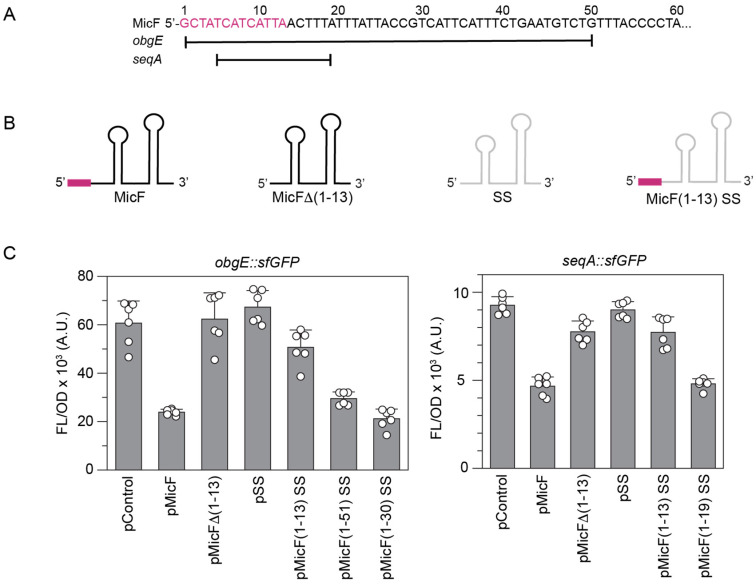
Identifying the base pair regions required for MicF’s regulation of *obgE* and *seqA*. (**A**) The sequence for the 5′ end of MicF and its seed pairing region (in magenta). Lines indicate the nucleotides predicted to interact with the mRNAs of *obgE* and *seqA*. A more detailed representation of the predicted interactions between *obgE* and *seqA* is displayed in Figure S5. (**B**) Schematics of the MicF variants. The magenta box represents MicF’s 13-nucleotide seed region. SS is the SgrS scaffold used for the testing of truncated MicF segments. (**C**) Bars show mean values of fluorescence/optical density (FL/OD_600_) from cells with *obgE::sfGFP* or *seqA::sfGFP* plasmids in the presence of a control plasmid (pControl) or one that overexpresses a MicF variant. Plasmids were transformed into *E. coli* BW25113. Error bars represent the standard deviations of six biological replicates, shown as open circles.

To further explore the binding between MicF and the two mRNAs, we examined the importance of MicF’s seed pairing region. A seed pairing region is an unstructured region that initiates base pairing with target mRNAs and is a feature of many sRNAs [38]. The seed pairing region of MicF consists of a conserved 13 nucleotides at the 5′ end that can fully regulate the mRNAs *lrp* and *ompF* when fused to an unrelated sRNA backbone [11,39]. To explore the role of MicF’s seed pairing region in the repression of *obgE* and *seqA*, we designed a synthetic sRNA composed of two parts: the first 13 nucleotides of MicF and the Hfq binding scaffold of an unrelated sRNA (SgrS) that has been used in the engineering of synthetic Hfq-dependent sRNAs [40,41,42] (Figure 5B). To test these constructs, we transformed *E. coli* BW25113 cells with plasmids expressing one of two MicF variants. The first is composed of the seed pairing region of MicF fused to the 5′ end of the SgrS scaffold (MicF(1–13) SS), and the second has the first 13 nucleotides of MicF deleted (MicF Δ(1–13)). The repression of both *obgE::sfGFP* and *seqA::sfGFP* was eliminated in the absence of MicF’s seed pairing region. However, the seed pairing region alone was not capable of fully repressing either gene (Figure 5C).

Next, we sought to identify the portion of MicF that would be sufficient for the repression of *obgE* and *seqA*. CopraRNA predicts that MicF binds to *obgE* in several different regions through nucleotide 51 of MicF, while only nucleotides 5–19 are predicted to interact with *seqA* (Figure S5). We built two new plasmid constructs where nucleotides 1–51 or 1–19 were fused to the 5′ end of the SgrS scaffold, pMicF(1–51) SS and pMicF(1–19) SS, respectively. While pMicF(1–19) SS sufficiently repressed *seqA::sfGFP*, pMicF(1–51) SS only moderately repressed *obgE::sfGFP* (Figure 5C). To further investigate *obgE* regulation, we considered the possibility that not all predicted interactions take place. CopraRNA does not account for Hfq binding, which is predicted to occur somewhere within the region that spans nucleotides 28 to 93 of MicF [43]. Thus, in MicF(1–51) SS, nucleotides 28–51 may interfere or compete with the SgrS scaffold and reduce its accessibility to Hfq. Since one way sRNAs regulate expression is through occlusion of the ribosome binding site (RBS), we used the RBS Calculator [44] to predict the RBS of *obgE* and fused the nucleotides of MicF that were predicted to bind through the RBS to the SgrS scaffold (pMicF(1–30) SS). Using this variant, we observed complete repression of *obgE::sfGFP* (Figure 5C), suggesting that regulation of *obgE* may occur through an interference with ribosome accessibility.

### 3.4. The Repression of obgE and seqA Is Not Influenced by RNase E Mediated Decay

While the first 19 nucleotides of MicF were sufficient to repress *seqA::sfGFP*, those nucleotides are predicted to bind within the CDS of *seqA*, downstream of both the RBS and start codon (Figure S5). This suggests an alternative mechanism for *seqA* repression that does not involve ribosome occlusion. Since sRNAs may control protein expression by modifying the stability of the mRNA transcript, we explored whether MicF exacerbates the decay of the *seqA* transcript by inducing its degradation by RNase E. In *Salmonella*, MicF represses *lpxR* through a dual mechanism involving the inhibition of ribosome accessibility and the stimulation of degradation by RNase E [11]. Therefore, we also probed MicF’s ability to induce the degradation of the *obgE* transcript by RNase E.

**Figure 6:**
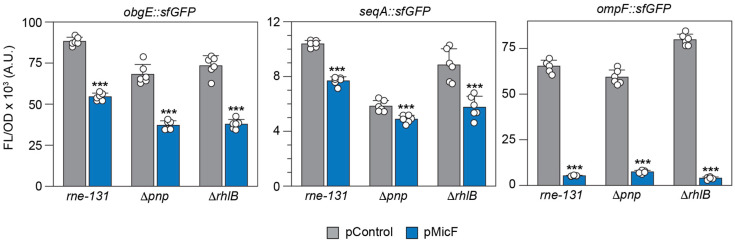
Contribution of the RNA degradosome in regulation by MicF. Bars show mean values of fluorescence/optical density (FL/OD_600_) from cells with *obgE::sfGFP*, *seqA::sfGFP*, or *ompF::sfGFP* plasmids in the presence of a control plasmid (pControl) or one that overexpresses MicF (pMicF). Plasmids were transformed into strains of *E. coli* BW25113 mutated to remove either the C-terminal half of RNase E (*rne-131*), PNPase (Δ*pnp*), or RhlB (Δ*rhlb*). Error bars represent the standard deviations of six biological replicates, shown as open circles. A two-tailed *t*-test was performed between the pControl and pMicF conditions. Significance is marked by asterisks above the pMicF bar, indicating *p* < 0.001 (***).

In *E. coli*, RNase E is one of the major enzymes involved in the formation of the multi-protein RNA degradosome. The N-terminal half of RNase E contains its endoribonuclease activity while the C-terminal half is natively unstructured and serves as a scaffold for the binding of the other degradosome components: PNPase, RhlB, and enolase [45]. The C-terminal domain of RNase E also interacts with Hfq, and this interaction is necessary for the sRNA-mediated degradation of mRNA [46]. To determine whether MicF’s repression of *obgE* and *seqA* involves Hfq-dependent RNase-E-mediated decay, we utilized a strain of *E. coli* where the entire C-terminal scaffold of RNase E is deleted (*rne-131*) [47,48]. Prior research has shown that MicF is stably expressed in the RNase-E-mutant strains *rne-131* [19] and *rne-701* [27]. The *rne-701* strain has another C-terminal truncation that prevents interaction with Hfq and degradosome assembly. In the *rne-131* strain, neither *obgE::sfGFP* nor *seqA::sfGFP* was fully repressed by MicF (Figure 6).

The *rne-131* truncation also prevents PNPase and RhlB from associating with RNase E. PNPase is a 3′ exoribonuclease, while RhlB is a DEAD-box RNA helicase. Both aid in the decay of the RNA intermediates created by RNase E and have reduced activity when not associated to RNase E [45]. Additionally, a complex between PNPase, Hfq, and the sRNA protects some sRNAs from degradation by RNase E [49,50]. Prior research demonstrated that the regulation of *ompF* by MicF does not depend on RNase E degradation [27]. We do observe a reduction in the fold repression of *ompF::sfGFP* in the *rne-131* strain (12-fold, Figure 6) as compared to the wild-type strain (30-fold, Figure S1). The effect seen on the regulation of all three mRNAs could be due to the reduced activity of PNPase and RhlB or the general reduction in RNA degradation associated with the *rne-131* strain. To assess this directly, we tested MicF regulation in strains that had an intact RNase E but deleted PNPase (Δ*pnp*) or RhlB (Δ*rhlb*).

If PNPase protects MicF from RNase E degradation, we would expect a decrease in MicF concentration and a reduction in regulation in the Δ*pnp* strain. Furthermore, we may observe an increase in FL/OD from the mRNA::sfGFP fusions due to a decrease in chromosomally expressed MicF. While we do observe a decrease in regulation for all three target mRNAs, we also observe a decrease in FL/OD from the mRNA::sfGFP fusions in the pControl condition (Figure 6). Since Hfq-RNA complexes compete for binding to RNase E with RhlB [46,51], in the Δ*rhlb* strain we would expect an increase in regulation if the mechanism were RNase E dependent. However, we observe a small decrease in regulation for all three target mRNAs (Figure 6). Together these results suggest RNase E is not involved in MicF’s repression of *obgE::sfGFP* and *seqA::sfGFP* and the observed reduction in regulation in the *rne-131* strain could be due to a general reduction in RNA degradation.

### 3.5. Chromosomally Expressed MicF Represses obgE and seqA sfGFP Fusions

**Figure 7:**
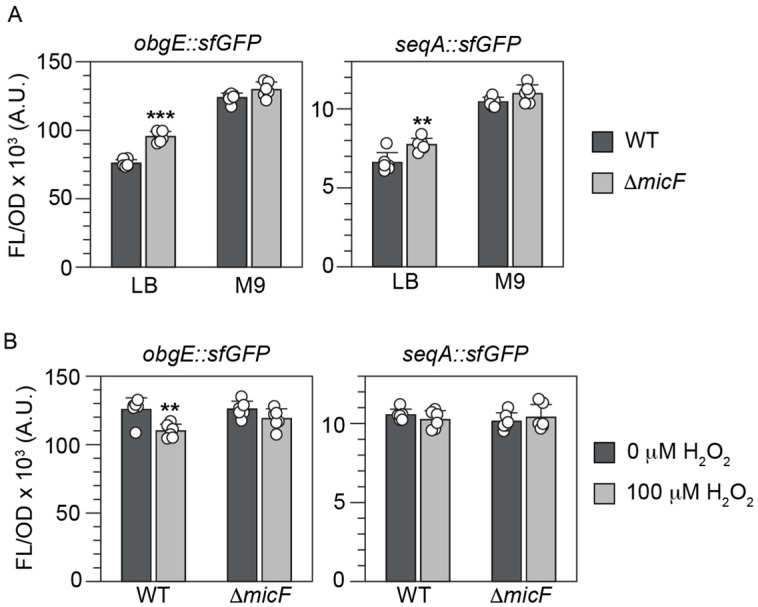
Effect of chromosomally expressed MicF on mRNA::sfGFP fusions. (**A**). *E. coli* BW25113 wild type (WT) or Δ*micF* was transformed with *obgE::sfGFP* or *seqA::sfGFP* plasmids and grown in LB or M9. (**B**) *E. coli* BW25113 wild type (WT) or Δ*micF* was transformed with *obgE::sfGFP* or *seqA::sfGFP* plasmids and grown in M9 with or without H_2_O_2_. Bars show the mean values of fluorescence/optical density (FL/OD_600_) and error bars represent the standard deviations of six biological replicates shown as open circles. A two-tailed *t*-test was performed between adjacent conditions in each graph. Significance is marked by asterisks above the light gray bar in the comparison indicating *p* < 0.01 (**) or *p* < 0.001 (***).

To evaluate the regulation of *obgE* and *seqA* without overexpressing MicF from a plasmid, we transformed our mRNA::sfGFP constructs into *E. coli* BW25113 wild-type cells or Δ*micF* cells and grew them under conditions known to vary in MicF concentrations. First, we took advantage of MicF being highly expressed in cells grown in nutrient-rich media such as LB and poorly expressed in cells grown in nutrient-poor media such as M9 [18,52]. For both *obgE::sfGFP* and *seqA::sfGFP*, we observe an increase in FL/OD in Δ*micF* cells when grown in LB as compared to wild-type cells. Furthermore, no change is observed in Δ*micF* cells when grown in M9 (Figure 7A).

### 3.6. The obgE sfGFP Fusion Is Repressed by MicF upon Exposure to Hydrogen Peroxide

The concentration of MicF is also known to increase in the presence of hydrogen peroxide due to activation by SoxRS [53]. To determine whether MicF influences *obgE* or *seqA* expression in response to hydrogen peroxide, we compared BW25113 wild-type and Δ*micF* cells transformed with the mRNA::sfGFP fusions when grown in M9 with or without hydrogen peroxide. A decrease in FL/OD was observed for *obgE::sfGFP* in the presence of hydrogen peroxide; however, no difference was noted for *seqA::sfGFP* (Figure 7B).

### 3.7. Overexpression of MicF Results in an Increase in Cell Doubling Time

In *E. coli*, the primary role of SeqA is to regulate the start of chromosome replication through its sequestration of hemimethylated origins of replication from the replication initiator protein DnaA [54]. Following replication, SeqA also aids in the cohesion of sister chromosomes prior to their segregation [55]. Mutants deficient in SeqA produce filamentous cells with unsegregated DNA and their doubling times are increased by approximately 20–30% [56,57]. To observe the effect of MicF overexpression, we measured the doubling time of several different strains (Table 1). An ANOVA was performed on the results, and the Holm method was used to control for the family-wise error rate (Table 2). Δ*micF* cells (no plasmid) and Δ*micF* cells complemented with a control plasmid (pControl) or a different sRNA (pMicC) had comparable doubling times to that of wild-type cells. Furthermore, Δ*micF* cells that overexpressed MicF (Δ*micF* pMicF) had increased doubling times when compared to the wild type. Although the average doubling time of Δ*micF* pMicF was not as high as that of the Δ*seqA* cells, the two data sets were not statistically different. To determine if the increase in doubling time was due to MicF’s repression of *seqA*, we simultaneously overexpressed MicF and SeqA from a plasmid in the Δ*micF* cells (Δ*micF* pMicF pSeqA). The overexpression of SeqA resulted in doubling times comparable to that of the wild type, thus linking the observed phenotype to MicF’s repression of *seqA*.

## 4. Discussion

Here, we experimentally validated two additional mRNA targets of MicF (*obgE* and *seqA*) that were predicted by CopraRNA. Notably, these two targets were also identified by MAPS [19]. ObgE is an essential GTPase in *E. coli* that has roles in chromosome partitioning [58,59] and in the cellular response to amino acid starvation [60]. SeqA serves as a negative modulator during the initiation of chromosome replication [54] that also facilitates the segregation of newly replicated sister chromosomes [55]. We found that MicF represses the expression of both genes.

Our experiments confirmed that MicF regulates both genes through an Hfq-dependent antisense mechanism that requires the 5′ seed pairing region of MicF. Unlike the regulation of OmpF and Lrp [11], the seed pairing region alone was not sufficient to repress *obgE::sfGFP* and *seqA::sfGFP* (Figure 5). The regulation of ObgE included the first 30 nucleotides of MicF that are predicted to bind through the RBS and start codon of *obgE*. The seed region itself is not involved in the pairing with the RBS or start codon (Figure S5), which suggests its role is to initiate binding between the two RNAs. Part of MicF’s seed pairing region is predicted to bind to *seqA*, although the first 19 nucleotides were required to completely repress *seqA::sfGFP*. In the case of SeqA, MicF is only predicted to bind within the CDS and not the RBS or start codon. It is still possible that MicF inhibits translation initiation even though it does not bind to the RBS or start codon. Prior research investigating RybB repression of *ompN* in *Salmonella* demonstrated that translational control could occur if base pairing took place within a five-codon window [61]. MicF is predicted to bind to the *seqA* CDS starting within the fourth codon.

**Figure 8:**
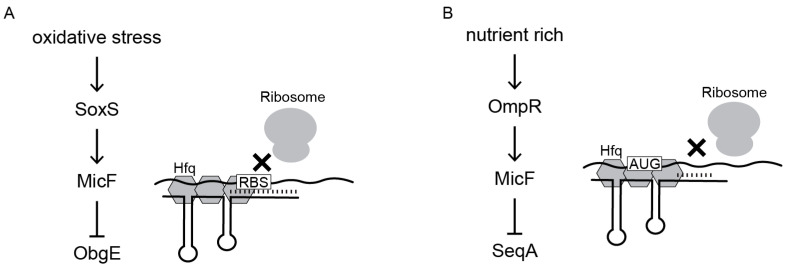
Proposed model for regulation of ObgE and SeqA by MicF. (**A**). Under oxidative stress MicF prevents translation of ObgE. (**B**). Under nutrient-rich conditions MicF prevents translation of SeqA.

Besides occluding translation initiation, sRNAs are known to repress protein expression by promoting mRNA decay by RNase E or RNase III [62,63]. RNase III is known to degrade dsRNA or intramolecular duplexes formed within a single RNA; however, in either case the duplex must be of sufficient length, approximately 22 base pairs [64]. In the case of *seqA*, the predicted binding interaction is only 15 base pairs long, which makes it a less likely target. We assessed the role of RNase E; however, neither the regulation of *seqA::sfGFP* nor *obgE::sfGFP* appeared to be dependent on RNase E (Figure 6). We acknowledge that the native mRNAs were not assessed and our results only provide insight to the mRNA-sfGFP fusions. Given these limitations, we propose that MicF represses the translation of both ObgE and SeqA by preventing the formation of the translation initiation complex (Figure 8).

The regulation of ObgE and SeqA introduces a new role for MicF beyond the regulation of the outer membrane. ObgE is known to play a critical role in chromosome partitioning and is potentially used in a replication checkpoint response [59,65]. Notably, chromosome partitioning defects have been observed with a modest reduction in ObgE concentrations [58]. Thus, repression of ObgE by MicF, although small, could contribute to cell cycle arrest. This helps to explain a role of MicF when activated by SoxS. Transcription of *soxS* is activated by SoxR, which is induced by superoxide-generating agents or nitric oxide [66]. Early work showed that MicF repressed OmpF due to the activation of the *soxRS* locus, although the importance of OmpF suppression under oxidative stress conditions was not clear [15]. Strains deficient in *soxRS* were shown to be hypersensitive to chloramphenicol and nalidixic acid, which does suggest a direct role for repression of OmpF, since one way these antibiotics enter the cell is through OmpF [67,68]. However, elimination of OmpF did not increase the resistance of *E. coli* to menadione, another agent known to induce *soxRS* [15]. Other work showed only a modest increase in Lrp expression in a Δ*micF* strain that was treated with paraquat [69]. Here, we observed repression of *obgE::sfGFP* in the presence of hydrogen peroxide (Figure 8A). Perhaps the role of MicF during oxidative stress is to suppress chromosome partitioning and work together with the OxyS sRNA to induce cell cycle arrest and provide time for DNA damage repair [70].

SeqA has also been tied to replication arrest; however, this occurs under conditions with high SeqA concentrations [71,72]. Instead, we propose a role for suppression of SeqA in high-nutrient conditions where MicF is already known to repress OmpF and Lrp [12,18]. Initiation of chromosome replication by DnaA is blocked by the binding of SeqA to hemimethylated *oriC*. This prevents immediate re-initiation and ensures a single round of replication per division cycle. However, under nutrient-rich conditions and faster growth rates it is known that *E. coli* can initiate a new round of replication before the previous round is complete [54,73]. The DnaA concentration increases with increasing growth rate [74] and a parallel decrease in SeqA concentration would aid in the initiation of another round of replication. Repression of SeqA under these conditions (Figure 8B) would complement MicF’s repression of OmpF and Lrp.

## Figures and Tables

**Table 1:** Average doubling time of *E. coli* variants ^1^.

Strain	DT (min) ± STD
Wild type (BW25113)	22.3 ± 1.6
Δ*seqA*	29.7 ± 2.5
Δ*micF*	23.9 ± 2.4
Δ*micF* pMicF	27.6 ± 1.8
Δ*micF* pMicF pSeqA	25.1 ± 0.6
Δ*micF* pControl	24.1 ± 1.9
Δ*micF* pMicC	24.1 ± 1.8

^1^ Data from six biological replicates.

**Table 2:** Pairwise *t*-test with Holm *p*-value correction for doubling time data in Table 1.

	Wild Type	Δ*seqA*	Δ*micF*	Δ*micF* pMicF	Δ*micF* pMicF pSeqA	Δ*micF* pControl
**Δ*seqA***	1.70 × 10^−6^ *					
**Δ*micF***	1	0.00014 *				
**Δ*micF* pMicF**	0.00036 *	0.76882	0.02492 *			
**Δ*micF* pMicF pSeqA**	0.97255	0.00024 *	1	0.03959 *		
**Δ*micF* pControl**	0.97255	0.00024 *	1	0.03959 *	1	
**Δ*micF* pMicC**	0.97255	0.00024 *	1	0.03959 *	1	1

* *p*-value < 0.05.

## Data Availability

Data is contained within the article or Supplementary Material. Dataset available on request from the authors.

## Supplementary Materials

The following supporting information can be downloaded at: https://www.mdpi.com/article/10.3390/microorganisms12122397/s1. This article contains supporting information: Table S1: Strains used in this study; Table S2: Important DNA sequences; Table S3: Plasmids used in this study; Table S4: Top 15 CopraRNA results; Table S5: Top 15 TargetRNA2 results, and Figure S1: FL/OD_600_ measurements for data in Figure 1; Figure S2: NUPACK RNA structure predictions; Figure S3: Regulation of *obgE::sfGFP* or *seqA::sfGFP* in a Δ*hfq* strain; Figure S4: Predicted secondary structure for *seqA-M1’::sfGFP*; Figure S5: CopraRNA predicted MicF-mRNA interaction for *obgE* and *seqA*. Ref. [75] can be found in Supplementary Materials.
