# Supplementary material for: The Small RNA MicF Represses ObgE and SeqA in Escherichia coli
Source: Microorganisms. 2024 Nov 22;12(12):2397. doi: 10.3390/microorganisms12122397 (PMC11676804; doi:10.3390/microorganisms12122397)
Supplement: Supplementary file 1 [file microorganisms-12-02397-s001.zip › microorganisms-3316039-supplementary.pdf]

Supporting Information for
The small RNA MicF represses ObgE and SeqA in *Escherichia coli*

Aaron Y. Stibelman[1,2], Amy Y. Sariles[1], Melissa K. Takahashi[1,*]

[1]Department of Biology, California State University Northridge, Northridge, CA 91330
[2]Department of Biosciences, Rice University, Houston, TX 77005
*corresponding author. Email: melissa.takahashi@csun.edu

Table of contents:

	**Description**	**Page**
Table S1	Strains used in this study	2
Table S2	Important DNA sequences	3-5
Table S3	Plasmids used in this study	6-7
Table S4	Top 15 CopraRNA results	7
Table S5	Top 15 TargetRNA2 results	8
Figure S1	FL/OD_{600} measurements for data in Figure 1	9
Figure S2	NUPACK RNA structure predictions	10
Figure S3	Regulation of *obgE::sfGFP* or *seqA::sfGFP* in a Δ*hfq* strain	11
Figure S4	Predicted secondary structure for *seqA-M1'::sfGFP*	11
Figure S5	CopraRNA predicted MicF-mRNA interaction for *obgE* and *seqA*	12
References		12

Table S1: Strains used in this study. Includes primers used to create strains made for this study.

Strain	Reference	Forward primer	Reverse primer
E. coli BW25113			
E. coli BW25113 Δ*hfq*	[75]		
E. coli BW25113 Δ*rhlB*	[75]		
E. coli BW25113 Δ*pnp*	[75]		
E. coli BW25113 Δ*micF*	This study	GTCAAAACAAAACCTTCACTCGCAACTAGAATAACTCCCGGTGTAGGCTGGAGCTGCTTC	AGTGTGTAAAGAAGGGTAAAAAAAACCGAATGCGAGGCATATGGGAATTAGCCATGGTCC
E. coli BW25113 *rne-131*	This study	CTTCTTCGGCGCACTGAAAGCGCTGTTCAGCGGTGGTTAAGTGTAGGCTGGAGCTGCTTC	GATTACTTTGAGCTAATTATTACTCAACAGGTTGCGGACGATGGGAATTAGCCATGGTCC
E. coli BW25113 Δ*rhlB* Δ*pnp*	This study	CGTAAGGTACTGTCTAAGAAAGAGAAAGGATATTACATTGGTGTAGGCTGGAGCTGCTTC	CGGAGGGCAAATGGCAACCTTACTCGCCCTGTTCAGCAGCATGGGAATTAGCCATGGTCC
E. coli BW25113 Δ*micF* *obgE*:3xFLAG	This study	CGAAGACGACGAAGAAGGCGTTGAGTTCATTTACAAGCGTGACTACAAAGACCATGACGG	AAATCGTGCAAATTCAACATATTGCAATTCTCTTGTAGGCATGGGAATTAGCCATGGTCC
E. coli BW25113 Δ*micF* *seqA*:3xFLAG	This study	ATTCCCGGCGGAATTGATTGAGAAGGTTTGCGGAACTATCGACTACAAAGACCATGACGG	TGGGCGACGTTAATCAAATCACTCTGTTGTGCAGGTTGGCATGGGAATTAGCCATGGTCC

Table S2: Important DNA sequences

**Name**	**Sequence**
J23118 promoter	TTGACGGCTAGCTCAGTCCTAGGTATTGTGCTAGC
J23119 promoter	TTGACAGCTAGCTCAGTCCTAGGTATAATACTAGT
Plux promoter	ACCTGTAGGATCGTACAGGTTTACGCAAGAAAATGGTTTGTTAT<br>AGTCGAATAAA
LuxR	ATGAAAAACATAAATGCCGACGACACATACAGAATAATTAATAA<br>AATTAAAGCTTGTAGAAGCAATAATGATATTAATCAATGCTTATC<br>TGATATGACTAAAATGGTACATTGTGAATATTATTTACTCGCGAT<br>CATTTATCCTCATTCTATGGTTAAATCTGATATTTCAATCCTAGA<br>TAATTACCCTAAAAAATGGAGGCAATATTATGATGACGCTAATTT<br>AATAAAATATGATCCTATAGTAGATTATTCTAACTCCAATCATTC<br>ACCAATTAATTGGAATATATTTGAAAACAATGCTGTAAATAAAAA<br>ATCTCCAAATGTAATTAAAGAAGCGAAAACATCAGGTCTTATCA<br>CTGGGTTTAGTTTCCCTATTCATACGGCTAACAATGGCTTCGGA<br>ATGCTTAGTTTTGCACATTCAGAAAAAGACAACTATATAGATAGT<br>TTATTTTTACATGCGTGTATGAACATACCATTAATTGTTCCTTCT<br>CTAGTTGATAATTATCGAAAAATAAATATAGCAAATAATAAATCA<br>AACAACGATTTAACCAAAAGAGAAAAAGAATGTTTAGCGTGGGC<br>ATGCGAAGGAAAAAGCTCTTGGGATATTTCAAAAATATTAGGTT<br>GCAGTGAGCGTACTGTCACTTTCCATTTAACCAATGCGCAAATG<br>AAACTCAATACAACAAACCGCTGCCAAAGTATTTCTAAAGCAAT<br>TTTAACAGGAGCAATTGATTGCCCATACTTTAAAAATTAA
T1 terminator	GCATCAAATAAAACGAAAGGCTCAGTCGAAAGACTGGGCCTTT<br>CGTTTTATCTGTTGTTTGTCGGTGAACGCTCTCCTGAGTAGGAC<br>AAATCCGCCGCCCTAGA
MicF	GCTATCATCATTAACTTTATTTATTACCGTCATTCATTTCTGAATG<br>TCTGTTTACCCCTATTTCAACCGGATGCCTCGCATTCGGTTTTTT<br>TT
SeqA CDS	ATGAAAACGATTGAAGTTGATGATGAACTCTACAGCTATATTGC<br>CAGCCACACTAAGCATATCGGCGAGAGCGCATCCGACATTTTA<br>CGGCGTATGTTGAAATTTTCCGCCGCATCACAGCCTGCTGCTC<br>CGGTGACGAAAGAGGTTCGCGTTGCGTCACCTGCTATCGTCGA<br>AGCGAAGCCGGTCAAAACGATTAAAGACAAGGTTCGCGCAATG<br>CGTGAACTTCTGCTTTCGGATGAATACGCAGAGCAAAAGCGAG<br>CGGTCAATCGCTTTATGCTGCTGTTGTCTACACTATATTCTCTTG<br>ACGCCCAGGCGTTTGCCGAAGCAACGGAATCGTTGCACGGTC<br>GTACACGCGTTTACTTTGCGGCAGATGAACAAACGCTGCTGAA<br>AAATGGTAATCAGACCAAGCCGAAACATGTGCCAGGCACGCCG<br>TATTGGGTGATCACCAACACCAACACCGGCCGTAAATGCAGCA<br>TGATCGAACACATCATGCAGTCGATGCAATTCCCGGCGGAATT<br>GATTGAGAAGGTTTGCGGAACTATCTAA
sfGFP<br>(no start codon)	AGCAAAGGAGAAGAACTTTTCACTGGAGTTGTCCCAATTCTTGT<br>TGAATTAGATGGTGATGTTAATGGGCACAAATTTTCTGTCCGTG<br>GAGAGGGTGAAGGTGATGCTACAAACGGAAAACTCACCCTTAA<br>ATTTATTTGCACTACTGGAAAACTACCTGTTCCGTGGCCAACAC<br>TTGTCACTACTCTGACCTATGGTGTTCAATGCTTTTCCCGTTATC<br>CGGATCACATGAAACGGCATGACTTTTTCAAGAGTGCCATGCC<br>CGAAGGTTATGTACAGGAACGCACTATATCTTTCAAAGATGACG<br>GGACCTACAAGACGCGTGCTGAAGTCAAGTTTGAAGGTGATAC

	CCTTGTTAATCGTATCGAGTTAAAGGGTATTGATTTTAAAGAAGA<br>TGGAAACATTCTTGGACACAAACTCGAGTACAACTTTAACTCAC<br>ACAATGTATACATCACGGCAGACAAACAAAAGAATGGAATCAAA<br>GCTAACTTCAAAATTCGCCACAACGTTGAAGATGGTTCCGTTCA<br>ACTAGCAGACCATTATCAACAAAATACTCCAATTGGCGATGGCC<br>CTGTCCTTTTACCAGACAACCATTACCTGTCGACACAATCTGTC<br>CTTTCGAAAGATCCCAACGAAAAGCGTGACCACATGGTCCTTCT<br>TGAGTTTGTAACTGCTGCTGGGATTACACATGGCATGGATGAG<br>CTCTACAAA
obgE (stop codon of previous gene through 40 codons)	GCATACAACGGTGGTATCGCAACCCCGCGCAGGCGAATGATTT<br>ACGGAGAATAAAATGAAGTTTGTTGATGAAGCATCGATTCTGGT<br>CGTTGCAGGTGATGGCGGTAATGGTTGCGTGAGCTTCCGCCG<br>CGAAAAGTATATTCCGAAAGGCGGCCCGGATGGCGGCGACGG<br>CGGTGATGGTGGTGACGTATGGATG
seqA (+1 through 40 codons)	ACTCCTGGCGACTTGTATTCAGCTAAGACACTGCACTGGATTAA<br>GATGAAAACGATTGAAGTTGATGATGAACTCTACAGCTATATTG<br>CCAGCCACACTAAGCATATCGGCGAGAGCGCATCCGACATTTT<br>ACGGCGTATGTTGAAATTTTCCGCCGCATCACAG
hofQ (30nt upstream of interaciton site through 40 codons)	ACTTAGCCAGTGGCGCTATCAGGGGATGGTAGGGCGAGGCGA<br>GCGCATCATCGGTGTAATAAAAGACGGGCAAAAGAAATGGCGA<br>CGGGTGCAGCAAAACGATGTGCTGGAAAACGGCTGGACAATTT<br>TACAGCTGACGCCAGACGTACTAACGCTGGGTACCGGGACAAA<br>CTGCGAACCGCCACAATGGTTGTGGCAACGGCAAGGAGATACA<br>AATGAAGCAATGGATAGCCGCACTACTGTTGATGCTGATACCC<br>GGCGTACAGGCGGCAAAGCCGCAAAAAGTGACGCTGATGGTG<br>GATGACGTTCCGGTAGCTCAGGTGTTGCAGGCGCTG
mgrB (+1 through 34 codons beyond interaction site)	ATAAGGTAGGTGAAACGGAGATTGGAGTGAAAAAGTTTCGATG<br>GGTCGTTCTGGTTGTCGTGGTGTTGGCTTGCTTGCTGCTTTGG<br>GCGCAGGTATTCAACATGATGTGCGATCAGGATGTACAATTTTT<br>CAGCGGAATTTGTGCCATTAACCAGTTTATCCCGTGG
hybB (+1 through 40 codons)	GAAGAACAAGAGGCCGAATGCTGGTGTGAAACATGCCAACAGT<br>ATGTGACGCTACTGACCCAGCGCGTCCGCCGCTGTCCACAGTG<br>TCATGGTGACATGCTGCAGATTGTGGCAGACGACGGTTTACAG<br>ATTCGGCGGATAGAAATAGACCAGGAGTGAGCGATGTGTACAA<br>CATGCGGTTGCGGTGAAGGCAACCTGTATATCGAGGGTGATGA<br>ACATAACCCTCATTCCGCGTTTCGTAGCGCGCCATTTGCCCCG<br>GCGGCACGCCCGAAGATGAAAATC
RBS-FlacZ	GAATTCATTAAAGAGGAGAAAGGTACCATGGACTACAAAGACCA<br>TGACGGTGATTATAAAGATCATGATATCGACTACAAAGATGACG<br>ACGATAAAACCATGATTACGGATTCACTGGCCGTCGTTTTACAA<br>CGTCGTGACTGGGAAAACCCTGGCGTTACCCAACTTAATCGCC<br>TTGCAGCACATCCCCCTTTCGCCAGCTGGCGTAATAGCGAAGA<br>GGCCCGCACCGATCGCCCTTCCCAACAGTTGCGCAGCCTGAAT<br>GGCGAATGGATGCAT
SgrS scaffold (SS)	TATTGGTGTAAAATCACCCGCCAGCAGATTATACCTGCTGGTTT<br>TTTTT
MicF(1-13)-SS	GCTATCATCATTATATTGGTGTAAAATCACCCGCCAGCAGATTA<br>TACCTGCTGGTTTTTTTT
MicF(1-19)-SS	GCTATCATCATTAACTTTATATTGGTGTAAAATCACCCGCCAGC<br>AGATTATACCTGCTGGTTTTTTTT

MicF(1-30)-SS	GCTATCATCATTAACTTTATTTATTACCGTTATTGGTGTAAAATCACCCGCCAGCAGATTATACCTGCTGGTTTTTTTT
MicF(1-51)-SS	GCTATCATCATTAACTTTATTTATTACCGTCATTCATTTCTGAATGTCTGTTATTGGTGTAAAATCACCCGCCAGCAGATTATACCTGCTGGTTTTTTTT
3xFLAG	GACTACAAAGACCATGACGGTGATTATAAAGATCATGATATCGACTACAAAGATGACGACGATAAA
obgE-M1’ (stop codon of previous gene through 5 codons)	TAAGCATACAACGGTGGTATCGCAACCCCGCGCAGGCGAATGATTTACGGAGAATAAAATGAAGTTTGTTGATcAAGCATCGATTCTGGTCGTT
obgE-M2’ (stop codon of previous gene through 5 codons)	TAAGCATACAACGGTGGTATCGCAACCCCGCGCAGGCGAATGATTTACGGAGAATAAAATGAAcTTTGTTGATGAAGCATCGATTCTGGTCGTT
seqA-M1’ (+1 through 20 codons)	ACTCCTGGCGACTTGTATTCAGCTAAGACACTGCACTGGATTAAGATGAAAACGATTGAAGTTGATGATcAACTCTACAGCTATATTGCCAGCCACACTAAGCAT
seqA-M2’ (+1 through 20 codons)	ACTCCTGGCGACTTGTATTCAGCTAAGACACTGCACTGGATTAAGATGAAAACGATTGAAcTTGATGATGAACTCTACAGCTATATTGCCAGCCACACTAAGCAT

Table S3: Plasmids used in this study

Plasmid	Description	Origin/Resistance	Figure
MKT176	J23118-T1	p15A/AmpR	1, 4, 5, 6, S1
MKT173	J23118-MicF-T1	p15A/AmpR	1, 4, 5, 6, S1
MKT221	J23118-T1	pSC101/CmR	1, 4, 5, 6, S1
MKT172	J23118-ompF(13 codons)-sfGFP-T1	pSC101/CmR	1, 6, S1
MKT702	J23119-lrp(20 codons)-sfGFP-T1	pSC101/CmR	1, S1
MKT602	J23118-RBS-FlacZ-obgE(5 codons)-sfGFP-T1	pSC101/CmR	1, 4, 5, 6, 7, S1
MKT603	J23118-RBS-FlacZ-obgE(10 codons)-sfGFP-T1	pSC101/CmR	1, S1
MKT604	J23118-RBS-FlacZ-obgE(20 codons)-sfGFP-T1	pSC101/CmR	1, S1
MKT605	J23118-RBS-FlacZ-obgE(40 codons)-sfGFP-T1	pSC101/CmR	1, S1
MKT419	J23118-seqA(5 codons)-sfGFP-T1	pSC101/CmR	1, S1
MKT420	J23118-seqA(10 codons)-sfGFP-T1	pSC101/CmR	1, S1
MKT421	J23118-seqA(20 codons)-sfGFP-T1	pSC101/CmR	1, 4, 5, 6, 7, S1
MKT437	J23118-seqA(40 codons)-sfGFP-T1	pSC101/CmR	1, S1
MKT524	J23118-RBS-FlacZ-hofQ(5 codons)-sfGFP-T1	pSC101/CmR	1, S1
MKT525	J23118-RBS-FlacZ-hofQ(10 codons)-sfGFP-T1	pSC101/CmR	1, S1
MKT526	J23118-RBS-FlacZ-hofQ(20 codons)-sfGFP-T1	pSC101/CmR	1, S1
MKT539	J23118-RBS-FlacZ-hofQ(40 codons)-sfGFP-T1	pSC101/CmR	1, S1
MKT401	J23118-mgrB(5 codons)-sfGFP-T1	pSC101/CmR	1, S1
MKT402	J23118-mgrB(10 codons)-sfGFP-T1	pSC101/CmR	1, S1
MKT403	J23118-mgrB(20 codons)-sfGFP-T1	pSC101/CmR	1, S1
MKT427	J23118-mgrB(34 codons)-sfGFP-T1	pSC101/CmR	1, S1
MKT447	J23118-hypB(5 codons)-sfGFP-T1	pSC101/CmR	1, S1
MKT425	J23118-hypB(10 codons)-sfGFP-T1	pSC101/CmR	1, S1
MKT426	J23118-hypB(20 codons)-sfGFP-T1	pSC101/CmR	1, S1
MKT448	J23118-hypB(40 codons)-sfGFP-T1	pSC101/CmR	1, S1
MKT707	J23118-RBS-FlacZ-obgE(5 codons)-M1'-sfGFP-T1	pSC101/CmR	4
MKT713	J23118-RBS-FlacZ-obgE(5 codons)-M2'-sfGFP-T1	pSC101/CmR	4
MKT709	J23118-seqA(20 codons)-M1'-sfGFP-T1	pSC101/CmR	4
MKT714	J23118-seqA(20 codons)-M2'-sfGFP-T1	pSC101/CmR	4
MKT704	J23118-MicF-M1-T1	p15A/AmpR	4
MKT715	J23118-MicF-M2-T1	p15A/AmpR	4
MKT433	J23118-MicFΔ(1-13)-T1	p15A/AmpR	5
MKT431	J23118-SgrS Scaffold (SS)-T1	p15A/AmpR	5
MKT432	J23118-MicF(1-13)-SS-T1	p15A/AmpR	5
MKT517	J23118-MicF(1-19)-SS-T1	p15A/AmpR	5
MKT572	J23118-MicF(1-30)-SS-T1	p15A/AmpR	5
MKT511	J23118-MicF(1-51)-SS-T1	p15A/AmpR	5

MKT046	J23119-T1	ColE1/KanR	2, T1
MKT050	J23119-MicF-T1	ColE1/KanR	2, T1
MKT058	J23119-MicC-T1	ColE1/KanR	T1
MKT306	J23119-T1	p15A/AmpR	2
MKT161	J23119-MicF-T1	p15A/AmpR	2
MKT628	J23119-MicF-T1-PluxR-seqA-T1	ColE1/KanR	T1
MKT731	PT7- seqA(20 codons)-sfGFP-T7terminator	ColE1/KanR	3
MKT732	PT7- RBS-FlacZ-obgE(5 codons)-sfGFP-T7terminator	ColE1/KanR	3
MKT049	PT7-MicF-T7terminator	ColE1/KanR	3
MKT055	PT7-Hfq-T7terminator	ColE1/KanR	3

Table S4: Top 15 CopraRNA results. Previously validated targets are highlighted in yellow.

**Rank**	**Gene Name**	**Gene Annotation**	**MicF predicted nucleotides**
1	*lrp*	leucine-responsive global transcriptional regulator	1 - 64
2	*oppA*	oligopeptide ABC transporter periplasmic binding protein	1 - 59
3	*murG*	N-acetylglucosaminyl transferase	1 - 51
4	*ompF*	outer membrane porin 1a (Ia;b;F)	1 - 33
5	*ysaB*	uncharacterized protein	3 - 54
6	*obgE*	GTPase involved in cell partitioning and DNA repair	1 - 51
7	*seqA*	negative modulator of initiation of replication	5 - 19
8	*hofQ*	DNA catabolic putative fimbrial transporter	27 - 88
9	*mgrB*	regulatory peptide for PhoPQ feedback inhibition	1 - 63
10	*hypB*	GTP hydrolase involved in nickel liganding into hydrogenases	1 - 53
11	*mobA*	molybdopterin-guanine dinucleotide synthase	15 - 59
12	*smrB*	putative DNA endonuclease	16 - 57
13	*wza*	colanic acid export protein; outer membrane auxillary lipoprotein	5 - 27
14	*hsdS*	specificity determinant for hsdM and hsdR	27 - 58
15	*gltL*	glutamate/aspartate ABC transporter ATPase	1 - 25

Table S5: Top 15 TargetRNA2 results

Rank	Gene Name	Gene Annotation	MicF predicted nucleotides
1	*sbcB*	exodeoxyribonuclease I; exonuclease I	23 - 39
2	*ppsA*	phosphoenolpyruvate synthase	59 - 74
3	*ylcG*	uncharacterized protein, DLP12 prophage	27 - 44
4	*metL*	Bifunctional aspartokinase/homoserine dehydrogenase 2	46 - 60
5	*ybaY*	outer membrane lipoprotein	29 - 42
6	*serA*	D-3-phosphoglycerate dehydrogenase	44 - 58
7	*nimR*	putative DNA-binding transcriptional regulator	61 - 75
8	*appB*	cytochrome bd-II oxidase, subunit II	45 - 60
9	*gltD*	glutamate synthase, 4Fe-4S protein, small subunit	46 - 60
10	*rmsD*	16S rRNA m(2)G966 methyltransferase, SAM-dependent	77 - 93
11	*gspD*	general secretory pathway component, cryptic	28 - 43
12	*ebgR*	transcriptional repressor	-8 - 7
13	*murI*	glutamate racemase	65 - 75
14	*ydjZ*	TVP38/TMEM64 family inner membrane protein	1 - 20
15	*lsrK*	autoinducer-2 (AI-2) kinase	44 - 54

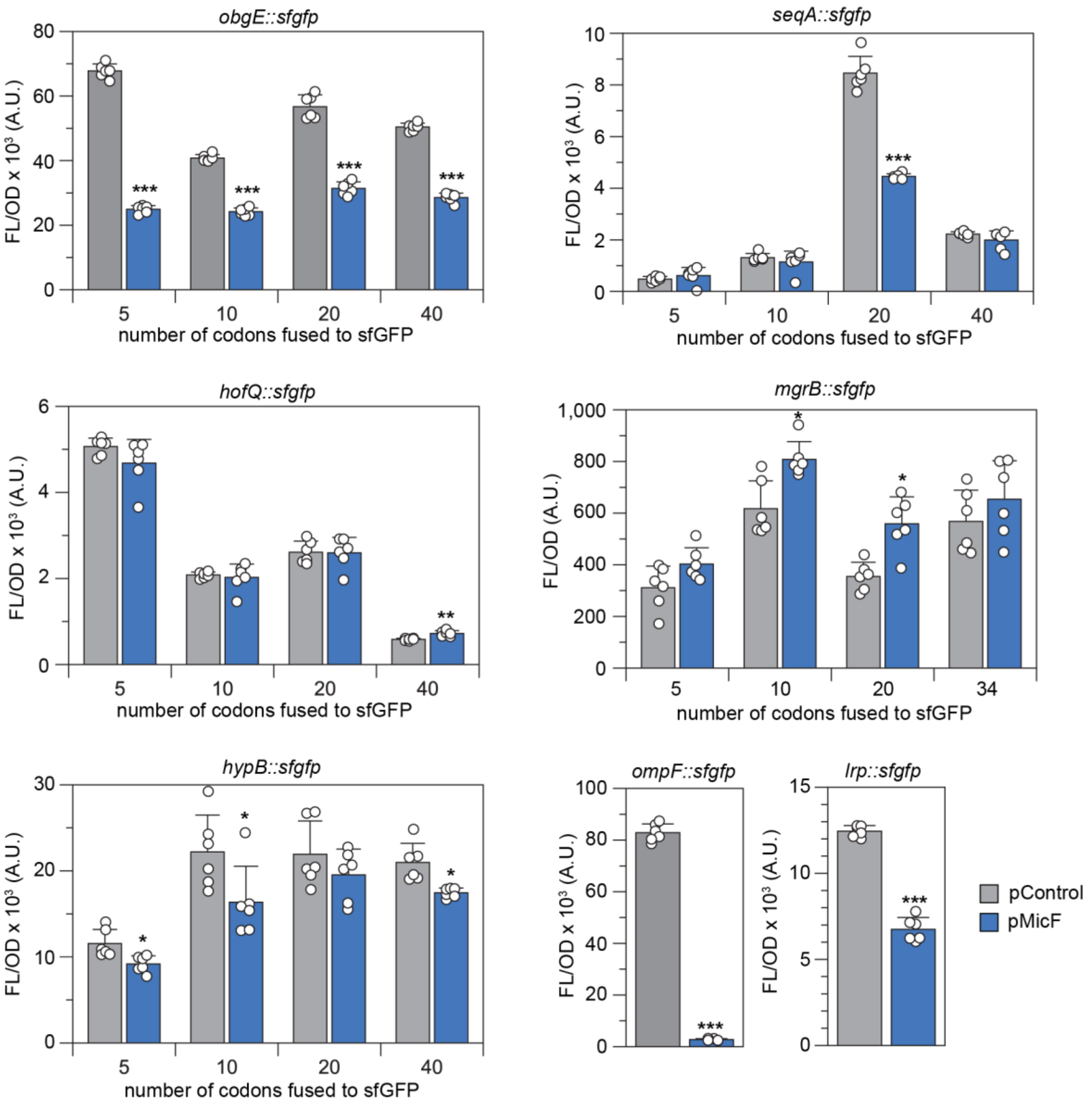

Figure S1. FL/OD_{600} measurements for data in Figure 1. Bars show mean values and error bars represent standard deviation of six biological replicates shown as open circles. Two-tailed t-tests assuming unequal variance were used, and the significance is marked by asterisks above the bars indicating $p < 0.05$ (*), $p < 0.01$ (**), $p < 0.001$ (***).

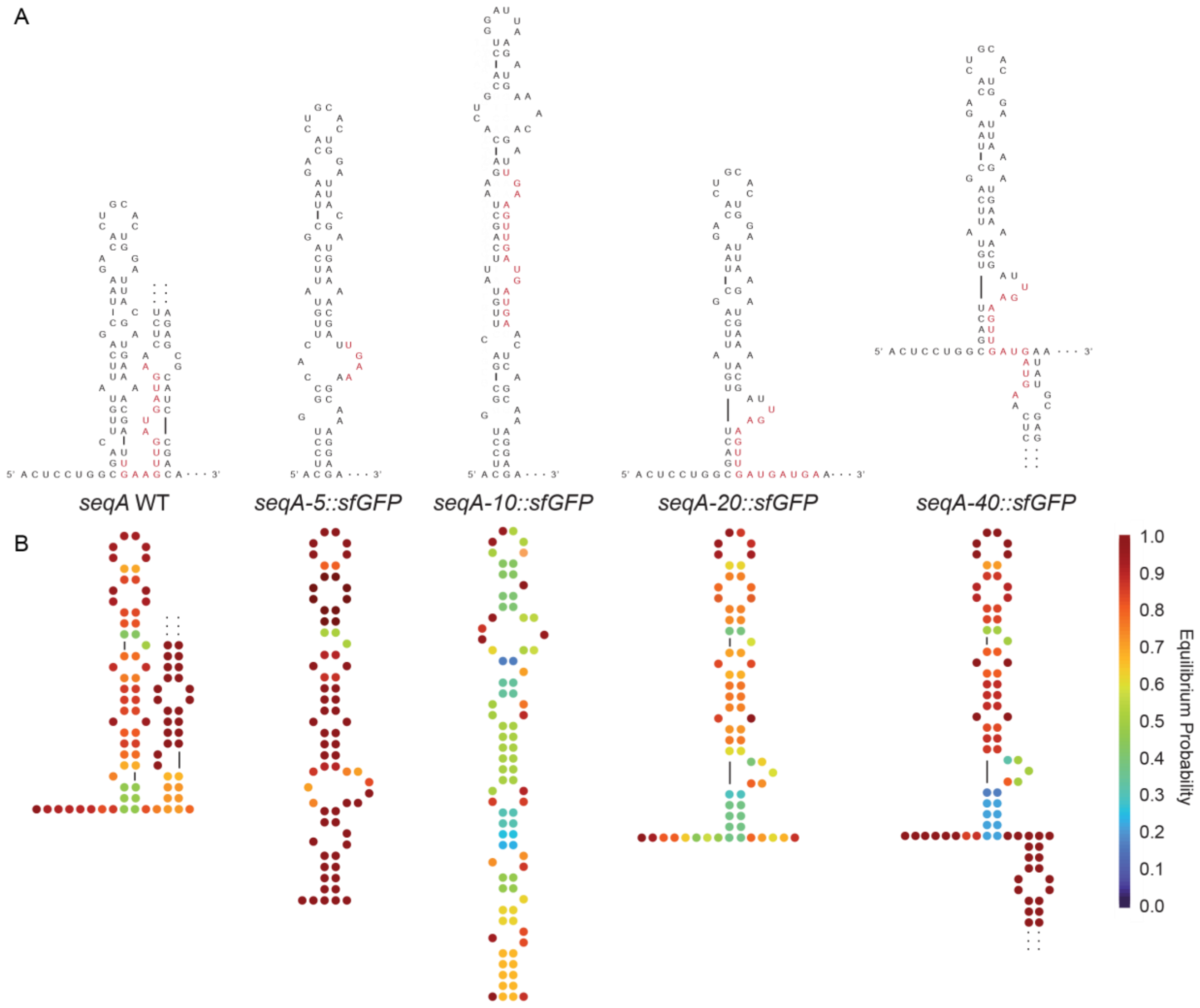

Figure S2. NUPACK RNA structure predictions. Secondary structures for *seqA::sfGFP* mRNA fusions were predicted using NUPACK. Sequences were folded in NUPACK and the region nearest the predicted MicF binding site is depicted here. A. Predicted secondary structures with the MicF binding site nucleotides colored in red. B. Equilibrium probability from NUPACK predictions for the nucleotides depicted in A.

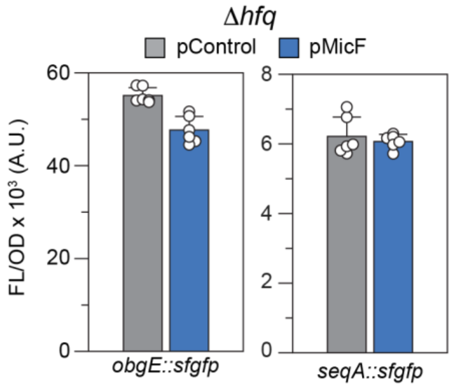

Figure S3. Regulation of *obgE::sfGFP* or *seqA::sfGFP* in a Δ*hfq* strain. Bars show mean values of FL/OD_{600} measurements and error bars represent standard deviation of six biological replicates shown as open circles.

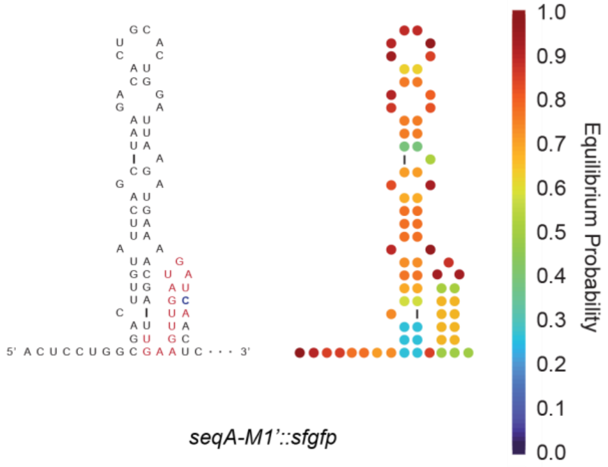

Figure S4. Predicted secondary structure for *seqA-M1'::sfGFP*. Secondary structure predicted by NUPACK with the MicF binding site nucleotides colored in red. Equilibrium probability predictions for the structure depicted on the right.

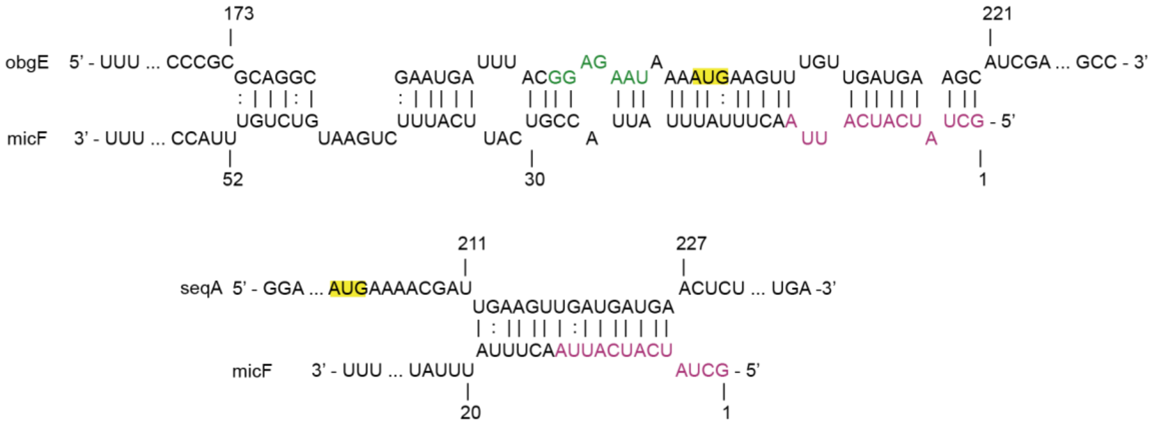

Figure S5. CopraRNA predicted MicF-mRNA interaction for *obgE* and *seqA.* Nucleotides in the predicted RBS of *obgE* are in green with the start codon highlighted in yellow. MicF's 13-nucleotide seed paring region is in magenta.

References

75. Baba, T.; Ara, T.; Hasegawa, M.; Takai, Y.; Okumura, Y.; Baba, M.; Datsenko, K.A.; Tomita, M.; Wanner, B.L.; Mori, H. Construction of Escherichia coli K-12 in-frame, single-gene knockout mutants: the Keio collection. *Mol. Syst. Biol.* **2006**, *2*, 2006-0008.
